# Cerebrospinal Fluid Creatine Kinase as a Biomarker in Dogs With Neurologic Disease: A Multivariate Analysis to Guide Future Directions

**DOI:** 10.1111/vcp.70126

**Published:** 2026-07-11

**Authors:** Taylor A. Hanson, Michael F. Rosser, Benjamin Blair, Graeme M. Doodnaught, Chandler C. Carter, Anne M. Barger, Kari Foss

**Affiliations:** ^1^ Department of Veterinary Clinical Medicine University of Illinois College of Veterinary Medicine Urbana Illinois USA; ^2^ Animal Health Partners Mississauga Ontario Canada; ^3^ Zoetis Reference Laboratories New Orleans LA USA

**Keywords:** canine, CK, CNS, CSF, spinal fluid

## Abstract

**Background:**

Creatine kinase (CK) is typically associated with skeletal muscle injury, but elevated CK in blood or cerebrospinal fluid (CSF) may also be reflective of central nervous system damage. Previous research has therefore explored the role of CK as a biomarker of neurologic disease in veterinary patients. However, the literature regarding this biomarker is somewhat incomplete, as control groups or reference populations have not always been included in previous studies.

**Objectives:**

This study aimed to further evaluate CSF CK in neurologic dogs—with a healthy control group for comparison—to investigate its predictive value for disease etiology and its relationship to neuroanatomic localization of disease, CSF collection site, other CSF parameters, and patient demographics.

**Animals:**

The control group included nine healthy Beagles. The neurologic cohort was comprised of 95 dogs with neurologic disease requiring CSF analysis.

**Methods:**

CSF was collected from appropriate sites with CK and routine parameters measured. A linear‐mixed effects model analyzed relationships between variables.

**Results:**

There was no significant difference in CSF CK between normal and neurologic dogs as a whole. No neurologic etiology differed significantly from other groups or from healthy controls except for dogs with metabolic disease (*n* = 2). Age and CSF total protein were significant predictors of CSF CK, but no significant relationship was identified between CSF CK and neuroanatomic localization or other parameters.

**Conclusions:**

We suspect that CSF CK is of limited utility in diagnostic differentiation or localization of neurologic disease in dogs. We suggest that future research explore alternative applications of this biomarker.

## 1 Introduction

Creatine kinase (CK) is an enzyme that catalyzes a reversible reaction between adenosine diphosphate (ADP) and phosphocreatine to adenosine triphosphate (ATP) and creatine to supply cellular energy in various tissues [1, 2]. CK can be released from injured cells, producing elevations that are detectable in the blood [2, 3, 4]. The major use of CK measurement in clinical practice is as a biomarker of skeletal muscle injury [4]; however, since it is also present in tissues of the central nervous system (CNS) [2, 5], there has also been much interest in whether CK may additionally provide insight about neurologic disease in veterinary species.

The association between neurologic disease and CK in veterinary species has been previously reported. For example, case reports have identified increased serum or cerebrospinal fluid (CSF) CK in a dog with necrotizing encephalitis [6], in calves and dogs with bacterial meningitis [7, 8], and in dogs with intervertebral disc disease [1]. CSF CK has also been investigated as a prognostic marker: for example, CSF CK was shown to be higher in dogs with thoracolumbar disc disease that had less successful return to function after injury [1]. There has also been interest in whether the degree of CSF CK activity might be reflective of disease etiology [4, 8, 9]. In this way, CSF CK might offer diagnostic benefit that could be of great use in veterinary medicine where other diagnostics are sometimes limited.

Several studies have been performed evaluating CK in relation to the etiologic category of neurologic disease. Paltrinieri et al. concluded that CK was not predictive of neurologic etiology in dogs, though this study investigated serum, rather than CSF CK [5]. Jackson et al. [3] evaluated CSF CK in horses and found that the magnitude of CSF CK was not able to differentiate between various neurologic diseases. Ferreira [9] conducted a similar study in 578 dogs with neurologic disease. Site of disease, etiology, and other variables were investigated [9]. Overall, the study revealed no relationship between CSF CK and patient breed, size, age, or sex, or between CSF CK and anatomic site or etiologic category of disease [5, 9]. However, this study was limited in that only cisternal samples were evaluated, even in cases where lumbar sampling would have been more appropriate to fully capture the patient's disease process [9]. Additionally, a control cohort of dogs was not included [9]. Only recently has a reference interval for CSF CK in dogs been proposed by Kopanke et al. [2] Considering the limitations and differing approaches of the studies present in the current literature, knowledge gaps surrounding this biomarker still persist.

Thus, we aimed to expand upon the existing knowledge of CSF CK as a biomarker in dogs, synthesizing previous approaches into a single study. We re‐evaluated the utility of CSF CK in diagnostic differentiation, its predictive value for neuroanatomic disease localization, and any association with other CSF parameters, collection site, or patient demographics.

## 2 Materials and Methods

### 2.1 Study Population and Inclusion Criteria

The study adhered to ethical standards and was approved by the Institutional Animal Care and Use Committee of the University of Illinois (protocol #22003). A population of Beagles maintained in a research colony served as the control cohort for this study. Dogs were included if they were greater than 1 year of age, had no history of neurologic or other disease, and were not currently receiving any medications other than standard parasite preventatives. Dogs received a routine general physical examination prior to being included in the study and must also have had a recent bloodwork analysis (including complete blood count and serum biochemistry profile) and urinalysis to exclude any obvious underlying disease.

Dogs with neurologic disease were recruited to the study between January 2022 and September 2022 and between October 2023 and April 2024. CSF samples were collected and analyzed during these time periods, and the medical records for each patient were later evaluated retrospectively during data compilation and statistical analysis. The neurologic cohort included dogs presenting to the Neurology and Neurosurgery Service of any age, breed, and sex with any suspected neurologic condition and any other medical history. All neurologic dogs underwent a complete physical and neurologic examination by a board‐certified veterinary neurologist or a resident in veterinary neurology under the supervision of a board‐certified neurologist. Dogs were not required to have any diagnostics other than CSF analysis in their medical record.

### 2.2 CSF Collection and Analysis

All dogs were placed under general anesthesia for CSF collection. For the healthy dogs, CSF collection was attempted from both the cerebellomedullary (CM) and lumbar sites by a veterinarian board‐certified in neurology following a standard technique [10]. CSF collection in the neurologic dogs was from either the CM or lumbar cistern. False negative results in CSF parameters, including CK, have been reported in dogs when CSF is not collected from the most appropriate site [11]; thus, whenever possible, CSF was collected from the cistern nearest—yet most caudal to—the lesion of interest based on neuroanatomic localization of clinical signs and any available imaging findings. Approximately 1 mL of CSF was collected into a sterile red‐top serum tube (Cardinal Health) and a sterile lavender‐top potassium‐EDTA tube (Covidien). CSF samples were analyzed within 30 min of collection by the Veterinary Diagnostic Laboratory at the University of Illinois.

Fluid analysis included total nucleated cell count (TNCC) in cells/mm^3^, red blood cell count (RBCs) in cells/mm^3^, and total protein (TP) concentration in mg/dL. TNCC and RBCs were obtained by manual count using a hemocytometer. TP was quantified via a standard biochemistry analyzer (Beckman Coulter DXC700AU, 2021) utilizing a microprotein assay designed for protein measurement within CSF or urine samples (urinary/CSF protein assay, BAOSR6X7013) [12]. CSF‐CK was quantified using an enzymatic activity assay in the same biochemistry analyzer (creatine kinase assay, BAOSR6X7913) [13]. CK activity is measured through a series of coupled enzymatic reactions that lead to the formation of NADPH. The rate of increase in absorbance at 340/660 nm, resulting from NADPH formation, is directly proportional to the CK activity in the sample [13]. Cytocentrifuged slides were also prepared from each CSF sample for review by a board‐certified clinical pathologist or a resident under supervision by a certified pathologist.

No limit was set for the maximum allowed number of total RBCs within CSF samples in order to be included, considering previous research that tends to favor no significant contribution from peripheral blood to CSF enzyme analysis in most cases [3, 9, 14, 15]. However, out of an abundance of caution, if the sample was interpreted cytologically by the pathologist as “hemodiluted sample,” “peripheral blood contamination,” or anything similar, that sample and patient were considered ineligible for the study to avoid overt contribution of serum CK to the total measured CK within the sample. Based on available data obtained for a few such samples, excluded samples typically referred to cases where the total RBCs were in the tens or hundreds of thousands and encompassed a variety of disease categories.

### 2.3 Classification of Neurologic Disease Etiology

After CSF collection and analysis were complete and data compiled, the medical record for each corresponding patient was retrospectively evaluated. Patients were classified into the following categories according to a commonly used etiologic scheme: “degenerative,” “anomalous,” “metabolic,” “neoplastic,” “inflammatory (non‐infectious),” or “infectious,” along with additional categories for “idiopathic epilepsy” and “unknown/other.”

Patients were categorized according to the most likely etiology of their presenting neurologic signs, based on: overall clinical suspicion inferred from the patient's neurologic examination, clinical signs, imaging studies, and other diagnostic test results (including infectious disease testing); in‐hospital treatment information; medical discharge notes; communication logs; and—when available—necropsy findings, response to therapy, and follow‐up visit notes. Infectious disease was considered the etiology if at least one diagnostic test returned a positive result for an infectious agent (e.g., bacterial culture or fungal antigen testing).

Additionally, patients were classified into the narrowest group when possible, based on presenting signs. For instance, a patient with two possibly relevant diagnoses of intervertebral disc disease (IVDD) and caudal occipital malformation syndrome (COMS) would be classified into the “degenerative” category for IVDD rather than “anomalous,” where other cases of COMS alone were placed. Most other cases of suspected congenital disease were placed into the “anomalous” category, and the “unknown/other” classification was used only for patients with an “open” diagnosis or where true neurologic disease was questionable (e.g., where behavioral changes were considered as a possible cause of the described neurologic signs).

### 2.4 Neuroanatomic Localization

Patients were also categorized by neuroanatomic localization of disease based on neurologic examination and in some cases, patient history. Categories included: “cerebrothalamus,” “central vestibular,” “cerebellum,” “brainstem,” “C1‐C5,” “C6‐T2”, “T3‐L3,” and “L4‐S3.” Patients were assigned to the most appropriate category based on their initial clinical neurologic examination, regardless of the exact location of any identified lesions. Patients were occasionally classified into a broader category such as “C1‐T2” and “T3‐S3” if the findings of the neurologic examination were more nebulous. Additionally, when the neurologic examination was suggestive of multifocal disease, patients were classified as “multifocal brain,” “multifocal spinal cord,” or “multifocal brain and spinal cord.”

### 2.5 Statistical Analysis

Data analyzed for each patient included age, breed, sex, site of CSF collection, etiology of neurologic disease, CSF CK, CSF TP, CSF TNCC, and CSF RBCs. Data processing and analysis were performed using Python (version 3.8) and associated libraries for statistical and graphical workup [16, 17, 18].

Descriptive statistics were used to report breed, age, sex, and site of CSF collection. The age of the patients was rounded down to the nearest year. For example, both “9.8 years old” and “9.1 years old” in the medical record would be recorded as “9 years old,” and patients less than 1 year old were recorded as “0 years old” to represent the age of the patient most appropriately while still facilitating numerical analysis. The mean and range were calculated for continuous variables (TNCC, RBCs, TP, and CK). Data normality was assessed using the Shapiro–Wilk test, and CK levels were log‐transformed where necessary to approximate normality.

A linear mixed‐effects model was constructed to adjust for potential confounders and to explore the relationship between CK levels and the other recorded variables. The fixed effects in the model included the etiologic category, breed, age, RBCs, TNCC, and TP, while the location of CSF sample collection was incorporated as a random effect to account for potential site‐specific variability. This multivariate approach allowed for the isolation of the independent effects of each variable on CK levels. Statistical significance was set at *p* < 0.05, and model fit was assessed through residual analysis and Akaike Information Criterion (AIC) values.

## 3 Results

### 3.1 Control Cohort

The healthy cohort consisted of 13 CSF samples from nine Beagles, including nine CM samples and four lumbar samples. The dogs ranged in age from 3 to 5 years, with a mean/median of about 4 years. The average CK value in the healthy cohort was approximately 112 ± 112 U/L (range: 13–338 U/L). When only CM samples were considered (for comparison to a recently proposed reference interval [2]), the average CSF CK value in our healthy cohort was about 128 ± 117 U/L (range: 38–338 U/L), with a median of 79 U/L.

The average values for RBCs, TNCC, and TP in the healthy cohort were 412 ± 678 cells/mm^3^ (range: 0–2370 cells/mm^3^), approximately 1 ± 1 cells/mm^3^ (range: 0–3 cells/mm^3^), and 18.6 ± 7.1 mg/dL (range: 9.7–33.3 mg/dL), respectively. Descriptive statistics for these parameters are also displayed for reference in Table 2 and Figure 2.

### 3.2 Neurologic Cohort

A total of 95 dogs with suspected neurologic disease met the inclusion criteria, with the dataset including 65 samples obtained from the CM cistern and 30 samples obtained from the lumbar cistern. The age of the dogs in the neurologic cohort ranged from less than 1 to 16 years, with a median of approximately 5 years. The neurologic cohort included dogs with the following disease etiologies: degenerative (*n* = 22), idiopathic epilepsy (*n* = 17), infectious (*n* = 12), inflammatory (*n* = 15), metabolic (*n* = 2), neoplastic (*n* = 10), anomalous (*n* = 9), and unknown/other (*n* = 8). The most commonly represented breeds in the neurologic cohort included mixed breed (*n* = 21), German Shepherd (*n* = 9), Labrador Retriever (*n* = 5), Pitbull breeds (*n* = 5), Beagle (*n* = 3), Cavalier King Charles Spaniel (*n* = 3), Chihuahua (*n* = 3), and Goldendoodle (*n* = 3), with 1–2 dogs included from a wide variety of other breeds.

The average CK value in dogs with neurologic disease overall was approximately 173 ± 206 U/L (range: 4–1189 U/L). The mean CSF CK level in the various groups of neurologic disease was as follows, in order of greatest to least: metabolic (696 ± 696 U/L, range: 204–1189 U/L), inflammatory (233 ± 244 U/L, range: 12–781 U/L), idiopathic epilepsy (185 ± 207 U/L, range: 4–655 U/L), infectious (157 ± 162 U/L, range: < 10–579 U/L), neoplastic (154 ± 146 U/L, range: 12–428 U/L), unknown/other (145 ± 140 U/L, range: 16–424 U/L), degenerative (143 ± 169 U/L, range: 6–692 U/L), and anomalous (74 ± 69 U/L, range: 9–189 U/L). These results are displayed in Figure 1 and Table 1.

**FIGURE 1:**
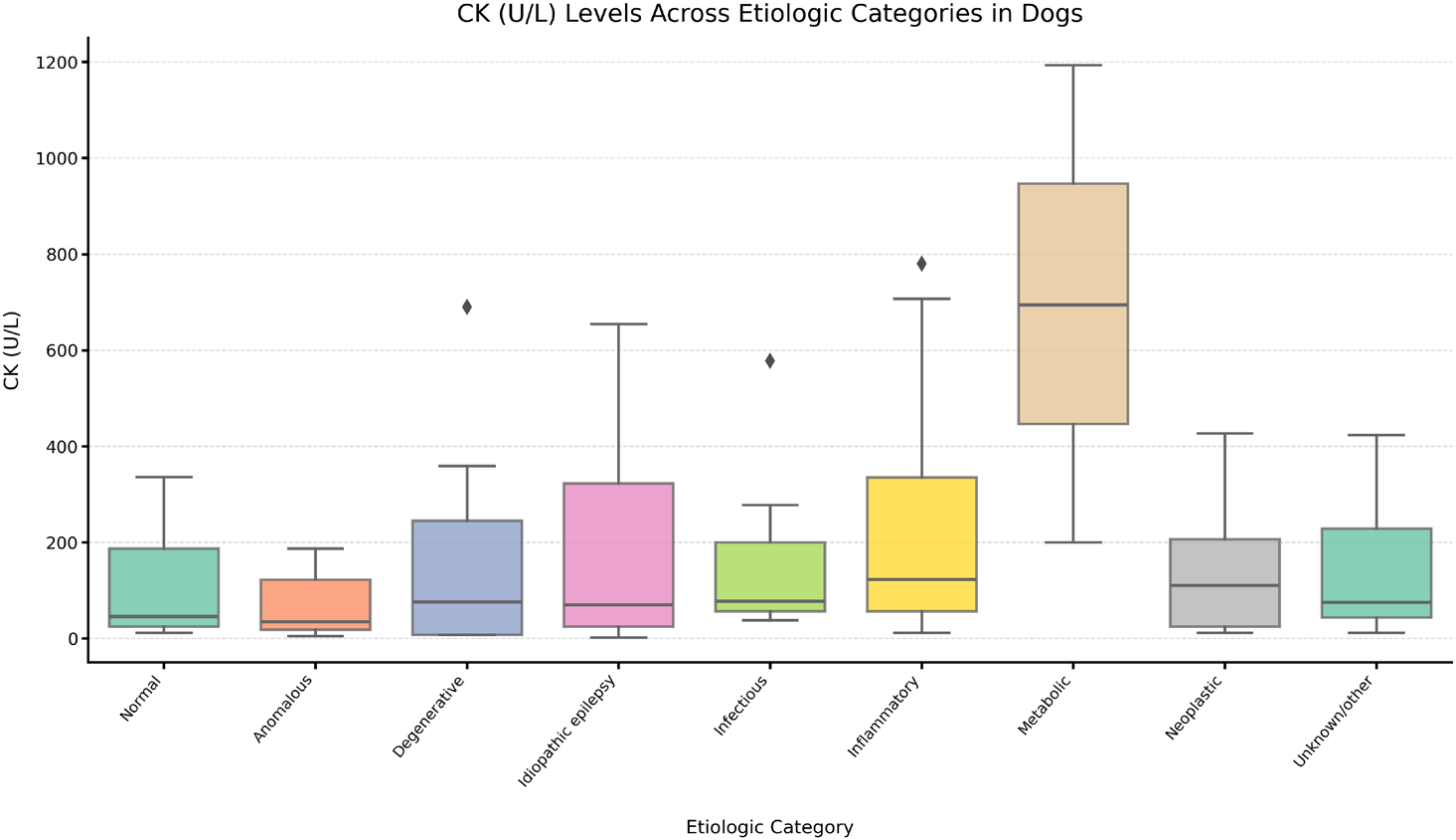
Box plot representing creatine kinase (CK) values in U/L within the cerebrospinal fluid of dogs (collected either via cerebellomedullary or lumbar cisternal tap) with the following etiologies of neurologic disease: Anomalous (*n* = 9), degenerative (*n* = 22), idiopathic epilepsy (*n* = 17), infectious (*n* = 12), inflammatory (*n* = 15), metabolic (*n* = 2), neoplastic (*n* = 10), and unknown/other (*n* = 8). A collection of samples from healthy dogs (“normal”) is also included (*n* = 13). For each category, the minimum and maximum values are represented by the vertical whiskers. Black diamonds represent possible outliers for any groups, where identified. Dogs with metabolic disease exhibited the highest mean CK level (696 U/L), which was significantly different than all other groups (*p* ≤ 0.001 in all cases).

**TABLE 1:** Descriptive statistics for cerebrospinal fluid (CSF) creatine kinase (CK) in dogs across all etiologic groups of neurologic disease.

Etiology	Mean CK	SD	Min	Max
Healthy (*n* = 13)	112	112	13	338
Anomalous (*n* = 9)	74.4	69.1	9	189
Degenerative (*n* = 22)	143.6	169.0	6	692
Idiopathic epilepsy (*n* = 17)	185.3	207.5	4	655
Infectious (*n* = 12)	157.5	162.6	< 10	579
Inflammatory (*n* = 15)	233.2	244.2	12	781
Metabolic* (*n* = 2)	696.5	696.5	204	1189
Neoplastic (*n* = 10)	154.4	146.6	12	428
Unknown/other (*n* = 8)	145.4	139.9	16	424

*Note:* The mean, standard deviation (SD), minimum value (min), and maximum value (max) for CK (in U/L) in each group are displayed. The only group that was significantly different from other groups, and from the healthy cohort, was metabolic disease (indicated with asterisk). No other etiologic groups had a significantly different CSF CK from each other or from the healthy cohort.

The average values for RBCs, TNCC, and TP in dogs with neurologic disease were 557 ± 1066 cells/mm^3^ (range: 0–6421 cells/mm^3^), 24 ± 104 cells/mm^3^ (range: 0–948 cells/mm^3^), and 63 ± 127 mg/dL (range: 10.7–1094 mg/dL), respectively. Descriptive statistics are displayed for reference in Figure 2 and Table 2.

**FIGURE 2:**
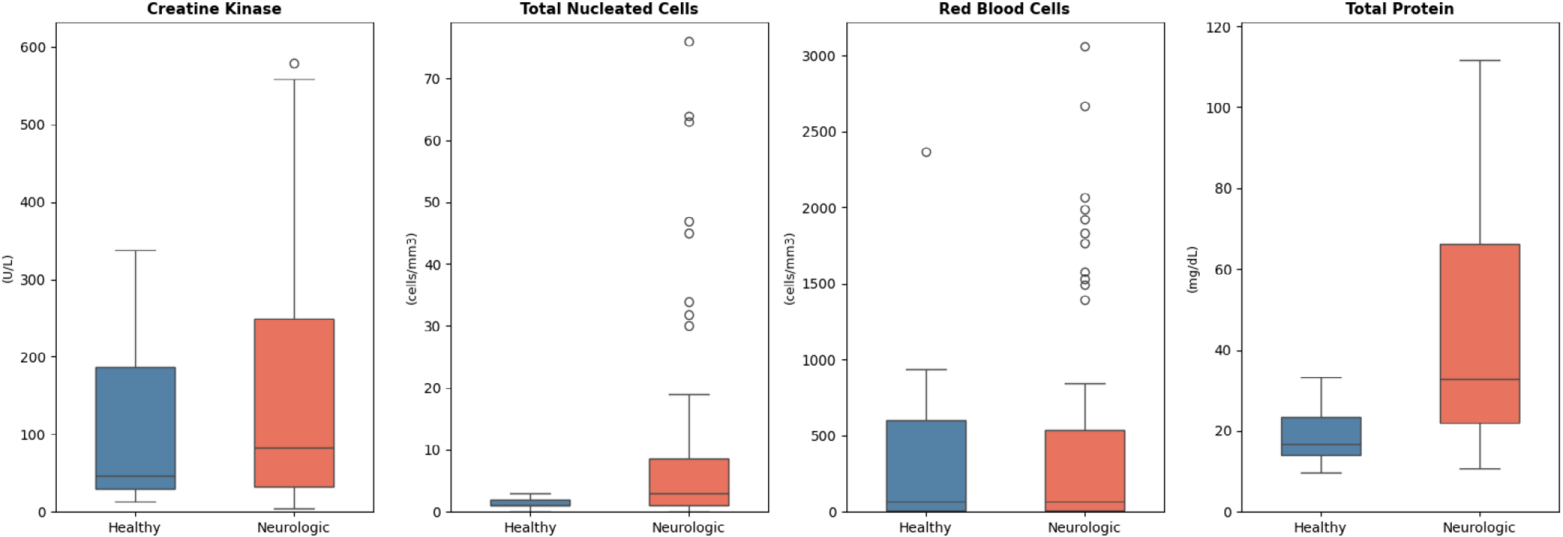
Distribution of selected cerebrospinal fluid parameters (creatine kinase in U/L, total nucleated cells in cells/mm^3^, total red blood cells in cells/mm^3^, and total protein in mg/dL) for both the neurologic (*n* = 95) and healthy (*n* = 13) cohorts are displayed. Values for neurologic dogs are displayed on the right for each parameter (shaded red), with values for healthy dogs displayed on the left for each parameter (shaded blue). Boxes represent the interquartile range (IQR, 25th to 75th percentile), median is shown as the horizontal line within each box, and whiskers extend to the most extreme values within 1.5 × IQR of the box edges. Outliers are shown as circles (though not all outliers are visualized because *y*‐axes are capped at the 95th percentile to adequately display the IQR and median while ensuring readability). Median values of the parameters are generally higher in the neurologic group compared to the control dogs, though these differences were not found to be statistically significant.

**TABLE 2:** Descriptive statistics for cerebrospinal fluid (CSF) parameters for both the neurologic (*n* = 95) and healthy (*n* = 13) cohorts.

Analyte	Group	Mean	Median	SD	Min	Max
Creatine kinase (U/L)	Neurologic	173	81	206	4	1189
	Healthy	111	46	112	13	338
Total nucleated cells (cells/mm^3^)	Neurologic	24	3	104	0	948
	Healthy	1	1	0.9	0	3
Red blood cells (cells/mm^3^)	Neurologic	557	65	1066	0	6421
	Healthy	412	65	678	0	2370
Total protein (mg/dL)	Neurologic	63.4	32.8	127.7	10.7	1094
	Healthy	18.6	16.7	7.1	9.7	33.3

*Note:* The mean, median, standard deviation (SD), minimum value (min), and maximum value (max) for creatine kinase (in U/L), total nucleated cells (in cells/mm^3^), red blood cells (in cells/mm^3^), and total protein (in mg/dL) are displayed. Mean and median values for nearly all parameters are higher in the neurologic cohort, including creatine kinase (CK). However, these values were not significantly different between the neurologic cohort at large and the healthy cohort, and CSF CK displays a wide range of values in both the neurologic and healthy cohorts. Total protein, however, was found to be a significant predictor of CSF CK (*p* = 0.05).

### 3.3 Comparison of Healthy and Neurologic Cohorts, and Relationship Between CSF CK and Other Variables

There was no significant difference in CSF CK, TP, TNCC, or RBCs between the neurologic dogs as a whole and the control group. However, statistical analysis using ANOVA (analysis of variance) demonstrated significant differences (*p* = 0.013) in CSF CK among some etiologic groups, and the post hoc Tukey's HSD (honestly significant difference) test rejected the null hypothesis in all instances where the metabolic group was compared to other groups. The metabolic category exhibited the highest mean CK level at 696 U/L, which was significantly different than all other categories of disease: anomalous (*p* = 0.0014), degenerative (*p* = 0.0034), idiopathic epilepsy (*p* = 0.0114), infectious (*p* = 0.0081), inflammatory (*p* = 0.0337), neoplastic (*p* = 0.0083), and unknown/other (*p* = 0.0087). This was also significantly different than the healthy cohort (*p* = 0.0023).

Additionally, the linear mixed‐effects model highlighted age (*p* = 0.02) and CSF TP (*p* = 0.05) as significant predictors of CK level. The relationships between age and CSF CK, and between CSF TP and CSF CK, are displayed in Figure 3. Furthermore, the sample collection location was included as a random effect in the model to account for any collection site‐specific variability. The model revealed a trend suggesting that CSF CK may differ between collection sites (with samples collected from the lumbar cistern possibly having higher CK), but no statistically significant differences were identified (*p* = 0.841). Breed and sex were not found to be significantly associated with CSF CK, nor were any other CSF analytical parameters (TNCC or RBCs).

**FIGURE 3:**
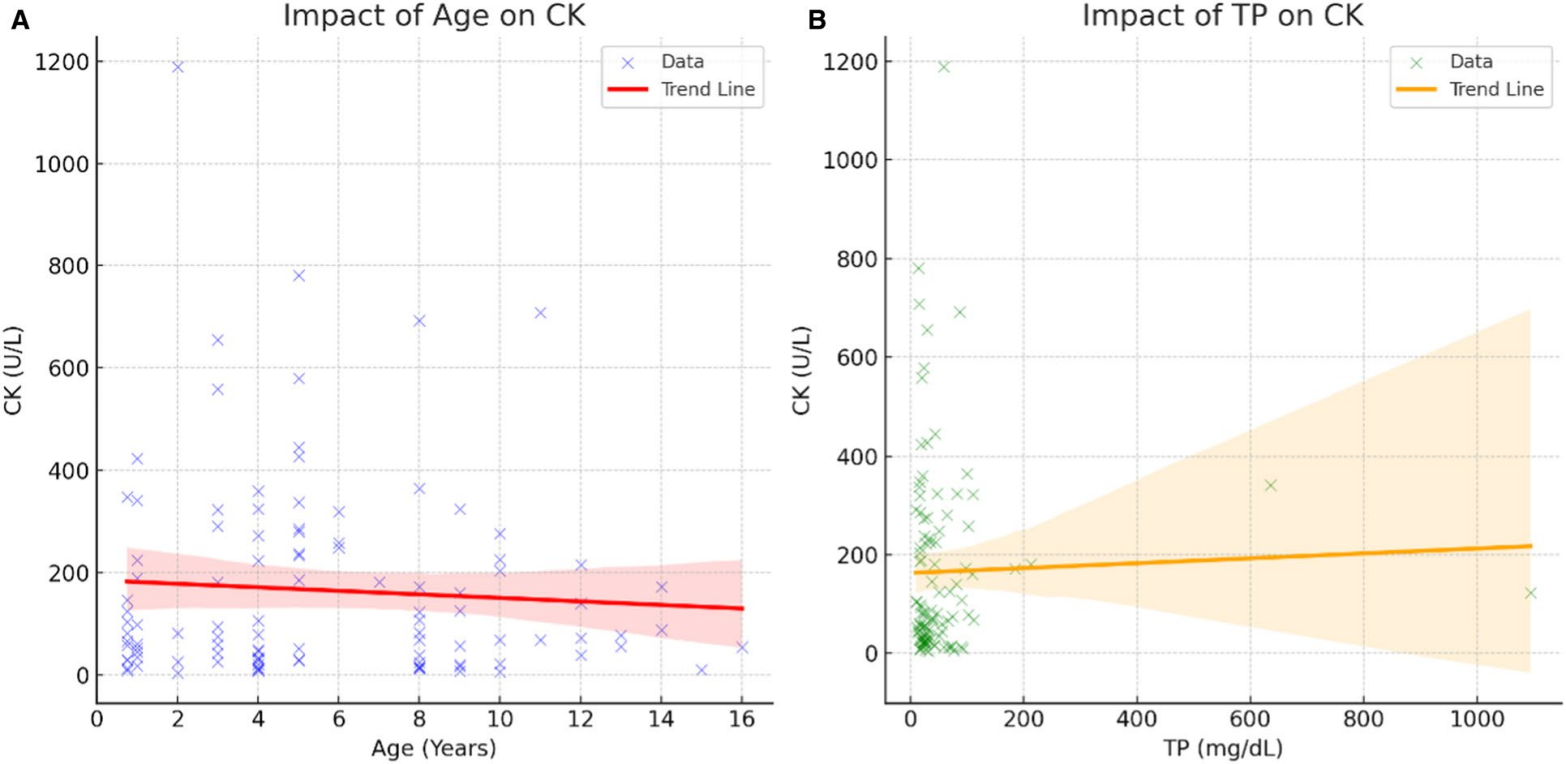
Age (A) and total protein concentration (TP) (B) as predictors of creatine kinase (CK) level in cerebrospinal fluid. Age was found to be a significant predictor of CK (*p* = 0.02), though the direction of this association is unclear as the 95% confidence interval (represented by the shaded region) covers both negative and positive associations. Considering the minimal negativity of the current trend line, it is possible that CK decreases to some degree with advancing age. TP was also found to be a significant predictor of CK (*p* = 0.05), though the direction of this association and its magnitude are also unclear as the 95% confidence interval (represented by the shaded region) covers a wide range of both negative and positive associations. Considering the mild positivity of the current trend line, especially at lower CK/TP values, it is possible that increases in TP correlate with mild increases in CK.

## 4 Discussion

Overall, CSF CK was not significantly increased in neurologic dogs as a whole compared to the control cohort. However, CSF CK was significantly increased in dogs with neurologic disease of metabolic origin compared to other etiologies. Among all patient signalment and CSF parameters, only patient age and CSF TP were significant predictors of CSF CK concentration. Additionally, CSF CK level did not correlate with any specific neuroanatomic localization of disease and was not significantly associated with CSF collection site.

The study found no statistically significant differences in CSF parameters between the neurologic cohort overall and normal dogs, even in TNCC or TP, which is an unexpected finding. The lack of significant differences between the two cohorts is suspected to be multifactorial. First, the neurologic cohort encompassed a wide variety of disease processes, including those that are expected to have cytologically unremarkable CSF (e.g., idiopathic epilepsy). Additionally, cytologically normal CSF samples do not exclude the presence of underlying neurologic disease; that is, cytologic evaluation and TP measurement within CSF do not always reflect the presence or severity of underlying neurologic disease. The fact that a wide variety of etiologic disease categories, including those expected to have normal or only mildly altered CSF findings, were grouped together in this cohort could contribute to the lack of significant differences as a whole compared with the normal dogs. Finally, given that only 13 samples were obtained from normal dogs, it is possible that the control group was not adequately powered to detect statistical differences in parameters like TNCC or TP compared to the neurologic group as a whole.

When the neurologic cohort was subdivided into etiologic categories of disease, significant differences were detected only among the metabolic category. Dogs with neurologic disease classified as metabolic in origin had a significantly higher CSF CK activity than normal dogs and all other etiologies of neurologic disease, though the sample size was very small. In this study, there were two dogs with neurologic disease classified as metabolic in etiology: one case of hypernatremia (sodium value 177 mmol/L, RI: 141–152 mmol/L) and one of hepatic encephalopathy. The highest CSF CK value documented in this study was identified in the dog with hypernatremia, at 1189 U/L.

The cause of possible CSF CK increase in metabolic conditions is unclear. In general, essentially three broad possibilities are posited to contribute, either in full or in part, to CK increases within the CSF: blood contamination during sampling, breakdown of the blood–brain or blood‐spinal cord barrier (such that the main source of CSF CK is of peripheral blood origin), or direct leakage of CK from neuroparenchyma secondary to cellular damage or dysfunction within the CNS [1]. The exact mechanisms underlying alterations in CSF CK activity in neurologic disease remain somewhat unclear, at least in veterinary species, and we speculate that they may vary based on the neuropathology present. At least in this study, the first proposition—that blood contamination during sampling represents a significant portion of CSF CK—seems unlikely to explain the observed significant increase in CSF CK in dogs with metabolic disease, as these dogs had relatively low evidence of peripheral blood contamination in their samples (0 and 20 red blood cells/mm^3^, with neurologic group mean of 557 red blood cells/mm^3^).

In particular, the association between metabolic conditions like hepatic encephalopathy and CSF CK activity has only rarely been addressed in the human medical literature, though there is speculation that increased brain ammonia and the resulting damage to astrocytes significantly alter cerebral energy metabolism [19, 20], which could then impact CK given its central role in neuronal energy production. Furthermore, in any severe metabolic or nutritional dysfunction affecting the CNS, cytotoxic edema of the brain is a possible sequela, which could cause neuronal injury and necrosis with subsequent leakage of cellular CK [21]. As disease progresses, intracranial edema could become so severe that localized ischemia or infarction occurs, which could then in fact disrupt the blood–brain barrier (BBB) and cause further CSF CK increases by allowing influx of systemic CK [21].

Certainly, this finding in dogs with metabolic derangements should be interpreted with caution, as only two dogs with neurologic disease classified as metabolic in origin were included in the study. Additional research with a larger sample size would therefore be required to confirm this finding, and to further evaluate what factors may contribute to any CSF CK elevation observed. However, it is dubious whether this finding—if confirmed—would be of significant clinical relevance, as a diagnosis is typically not elusive in patients with metabolic derangements severe enough to incite neurologic disease; in these cases, a routine biochemical analysis often reveals the etiology of the clinical signs, and additional diagnostic markers are likely not clinically necessary.

This study also attempted to investigate whether total CSF CK levels were significantly associated with neuroanatomic localization of disease. Researchers have previously speculated that the level of CSF CK activity may be dependent on the location of the primary neurologic disease process [22]. However, the mixed‐effects statistical model developed in this study did not identify neuroanatomic localization of CNS pathology as a significant predictor of CSF CK value. It therefore seems unlikely that the magnitude of CSF CK activity alone could be a predictor of lesion location in cases where clinical signs are vague or localize to more than one area.

CSF TP was found to be a significant predictor of CSF CK activity (*p* = 0.05). Because the 95% confidence interval covers both negative and positive associations, the exact relationship between these two variables is somewhat unclear (Figure 3B). However, because the current trend line of the data is mildly positive (especially at lower CK/TP values, which were more common in the dataset), it is likely that CSF CK increases alongside CSF TP (Figure 3B). In that case, this finding could support the idea that BBB compromise might indeed be a factor contributing to increasing levels of CSF CK (since increased CSF TP is often associated with BBB damage), with not all CSF CK identified in cases of neurologic disease being attributable to neuronal injury within the CNS.

Finally, this study also identified patient age as a significant predictor of CSF CK level (*p* = 0.02), which was an unexpected finding. The magnitude and direction of this association are again unclear, as the 95% confidence interval covers both negative and positive associations (Figure 3A). However, the current best‐fit trend line is slightly below zero, suggesting a minimal decrease in CSF CK with advancing patient age (Figure 3A).

The current literature regarding the relationship between CSF CK and patient age is sparse. It has long been generally accepted knowledge that serum CK tends to decrease with patient age [4, 23]. However, it has not been clearly demonstrated whether the same trend can be expected of CSF CK activity. Ferreira did not identify any association between CSF CK and age of dogs in their study [9]; however, in Kopanke et al.'s reference interval study, the highest CSF CK values were recorded in the two youngest dogs in the cohort [2]. The possibility that CSF CK could partially be dependent on patient age is important to consider, since age could be a confounding factor if not appropriately accounted for in studies involving CSF CK or when comparing CSF CK of neurologic patients to published reference intervals.

In the Kopanke et al. study, a median CSF CK of 3.7 U/L (range: 2.0–84.0 U/L) was reported in samples obtained from the CM cistern in healthy dogs [2]. Of note, the median CSF CK value in the healthy cohort in our study (including only CM samples) was higher at 79 U/L, and with a wider range of 38–338 U/L. Reference intervals are expected to vary somewhat between instruments and laboratories. However, the discrepancy between our respective findings seems subjectively higher than would be expected from standard analytical variation, especially as the biochemical analyzer used in the Kopanke et al. study utilizes a similar methodology to that of the Beckman Coulter instrument employed in this study [2]. A likely contributing factor is the small, demographically limited cohort of the present study, which is probably an insufficient representation of the patient population described in Kopanke et al.'s publication. Nonetheless, this finding highlights the importance of establishing laboratory‐specific reference values for CSF CK.

## 5 Study Limitations

Although a total of 95 dogs with neurologic disease were evaluated, disease etiology was split into eight separate categories, resulting in groups as small as only two patients. Thus, some of the etiologic groupings were likely underpowered, which could result in a lack of identification of significant differences in CSF CK. Alternatively, in the case of dogs with metabolic disease, the very small sample size could have resulted in erroneous rejection of the null hypothesis (Type I error)—especially considering that one of these dogs displayed the highest CSF CK value documented (nearly 400 units higher than the second‐highest value). This single value—which might theoretically represent an outlier or even an analytical error—could therefore have skewed the results significantly.

Additionally, the normal cohort of dogs that was used for statistical comparison in this study consisted of only 13 samples from nine individuals. Although the CSF CK values from this cohort did appear to visually approximate a baseline to some degree (Figure 1), a larger and more diverse cohort of normal individuals would have been preferred to better approximate the demographics of the neurologic cohort, optimize statistical analysis, or even be used as an institutional reference population. Ultimately, however, the control cohort was limited due to ethical considerations regarding CSF collection in healthy dogs. Furthermore, the normal cohort consisted only of apparently healthy dogs with no obvious evidence of neurologic or other disease; however, because a complete neurologic examination was not performed on these dogs, it cannot be entirely excluded that more occult neurologic disease may have been present in any given individual.

It is, of course, possible that our measured CSF CK values were somewhat impacted by the complexity involved in categorizing some cases, as well as the timing of sample collection. Some dogs had only a suspected diagnosis, or presented with clinical signs that may have localized to more than one neuroanatomic location, and definitive evaluation via tissue biopsy or necropsy was available only in a very small minority of cases. Furthermore, the timing of CSF collection after onset of clinical signs was not standardized since the optimal time to detect CSF CK elevations has not yet been established for various disease processes in dogs [9]. However, CK is considered fairly transient, as it is released from cells following injury and cleared from the CSF relatively rapidly [24]. We must therefore consider that peaks in CSF CK were not always documented in this study, which could have confounded results.

Finally, the enzymatic activity assay used to measure CK has not been independently validated in a peer‐reviewed publication for use in dogs, either for CSF or serum samples. Although the laboratory performs quality control and calibration daily for this assay using two levels of commercially available controls and calibration material, the application in a species for which the assay system is not validated must be noted.

## 6 Conclusion

Dogs with metabolic disease were the only group with higher CSF CK activity than the healthy dogs and other categories of disease, though only two patients with metabolic disease were present in the study. This finding should therefore be interpreted with caution and may in fact be of little clinical utility as the etiology of neurologic signs is typically easily recognized in these cases. Patient age and CSF TP were found to be significant predictors of CSF CK level. While these findings raise interesting questions about the factors that influence CSF CK in dogs with neurologic disease, these are also likely of low clinical utility.

In conclusion, considering the results of this study and the existing veterinary literature, we suggest that measurement of CSF CK is likely not clinically useful to determine etiology of neurologic disease, refine neuroanatomic localization, or predict other CSF parameters. We propose that any further research into CSF CK in dogs explore other applications—such as prognostication of specific disease processes, for example—rather than routine diagnostic utility.

## Conflicts of Interest

The authors declare no conflicts of interest.
